# Partial pressure of oxygen in adipose tissue and its relationship with fatness in a natural animal model of extreme fat deposition, the grey seal

**DOI:** 10.14814/phy2.14972

**Published:** 2021-08-19

**Authors:** Laura Oller, Kimberley A. Bennett, J. Chris McKnight, Simon E.W. Moss, Ryan Milne, Ailsa J. Hall, Joel Rocha

**Affiliations:** ^1^ Division of Health Sciences School of Applied Sciences Abertay University Dundee UK; ^2^ Sea Mammal Research Unit Scottish Oceans Institute University of St Andrews St Andrews UK; ^3^ Division of Sports and Exercise Sciences School of Applied Sciences Abertay University Dundee UK

**Keywords:** adiposity, blubber, marine mammals, NIRS, oxygenation, Po_2_, temperature

## Abstract

Excessive adiposity is associated with altered oxygen tension and comorbidities in humans. In contrast, marine mammals have high adiposity with no apparent detrimental effects. However, partial pressure of oxygen (Po_2_) in their subcutaneous adipose tissue (blubber) and its relationship with fatness have not been reported. We measured Po_2_ and temperature at different blubber depths in 12 healthy juvenile grey seals. Fatness was estimated from blubber thickness and morphometric parameters. Simultaneously, we monitored breathing pattern; heart rate and arterial blood saturation with a pulse oximeter; and relative changes in total hemoglobin, deoxyhemoglobin, and oxyhemoglobin in blubber capillaries using near‐infrared spectroscopy (NIRS) as proxies for local oxygenation changes. Blubber Po_2_ ranged from 14.5 to 71.4 mmHg (39.2 ± 14.1 mmHg), which is similar to values reported in other species. Blubber Po_2_ was strongly and negatively associated with fatness (LME: *p* < 0.0001, *R* ^2^_marginal_ = 0.53, *R* ^2^_conditional_ = 0.64, *n* = 10), but not with blubber depth. No other parameters explained variability in Po_2_, suggesting arterial blood and local oxygen delivery did not vary within and between measurements. The fall in blubber Po_2_ with increased fatness in seals is consistent with other animal models of rapid fat deposition. However, the Po_2_ levels at which blubber becomes hypoxic and consequences of low blubber Po_2_ for its health and function, particularly in very fat individuals, remain unknown. How seals avoid detrimental effects of low oxygen tension in adipose tissue, despite their high and fluctuating adiposity, is a fruitful avenue to explore.

## Graphical abstract

Grey seals blubber Po_2_ is similar to that measured in other mammal species and negatively associated with fatness, independently of depth of measurement, oxygen delivery, or temperature.

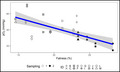

**Key points**

- Low oxygen levels in adipose tissue, as a result of tissue expansion, have been linked to poor metabolic health in obesity studies.
- Seals have a thick subcutaneous fat layer, called blubber. Blubber Po_2_ and its relation with fatness or tissue expansion has never been studied.
- Grey seals blubber Po_2_ was similar to that measured in other mammal species.
- Blubber Po_2_ was negatively associated with fatness, independently of depth of measurement, oxygen delivery, or temperature.
- Our results suggest that adipose tissue expansion lowers Po_2_, not only in rodents and humans, but in a wild mammal species that undergoes rapid fat deposition.

## 1 INTRODUCTION

Obesity is a growing problem for human health. Obesity has doubled its prevalence worldwide in the last 30 years, costing the UK £27 billion a year (Public Health England, 2017; World Health Organisation, 2020). Obesity is associated with the development of comorbidities that are linked to low oxygenation in adipose tissue, which can lead to oxidative stress and inflammation (Hosogai et al., 2007; Trayhurn, 2013; Wang et al., 2007).

Hypoxia, a deficiency in tissue oxygen levels, results from an imbalance between oxygen (O_2_) demand and supply (Rausch et al., 2008). In adipose tissue, hypoxia has been proposed to result from insufficient angiogenesis (Goossens et al., 2011; Pasarica et al., 2009) and/or reduced supply of oxygen (Trayhurn, 2013) as tissue expands. Indeed, many studies have consistently reported reduced blood flow to adipose tissue in obese humans (Frayn & Humphreys, 2011; Goossens et al., 2011; Vink et al., 2017).

While these findings suggest that adipose tissue becomes hypoxic as it expands, direct measurements of partial pressure of oxygen (Po_2_) are needed to confirm if reduced oxygen supply results in reduced oxygen availability. Studies investigating adipose tissue Po_2_ in rodents report a significant decrease of O_2_ availability with increased fatness as well as increased expression of hypoxia‐related genes, such as hypoxia‐inducible transcription factor‐1 alpha (HIF‐1α) (Rausch et al., 2008; Ye et al., 2007). However, results in humans are inconsistent, with studies reporting similar (Hiltebrand et al., 2008; Kaiser et al., 2016), lower (Cifarelli et al., 2020; Kabon et al., 2004; Lawler et al., 2016; Pasarica et al., 2009), and higher (Goossens et al., 2011, 2018) adipose tissue Po_2_ in obese compared to lean individuals. In addition, Vink et al. (2017) report a decrease in adipose tissue Po_2_ after diet‐induced weight loss. These conflicting findings may be partly explained by the variety and complexity of methods used to measure Po_2_, as well as the intrinsic features of each study species (Cifarelli et al., 2020; Hodson et al., 2013; Lempesis et al., 2019). Lempesis et al. (2019) suggest that rodent models of obesity are not comparable to human obesity since their rapid adipose tissue gain reflect artificial experimental conditions and not natural development of adipose stores. Numerous mammal species routinely undergo large and rapid fat accumulation as a normal part of their life cycle, without any apparent detrimental consequences (Khudyakov et al., 2019). Important insights may be gained from investigation of oxygen management and changes in Po_2_ during rapid fat accumulation in these species.

Marine mammals accumulate fat in specialized subcutaneous adipose tissue, called blubber. Apart from its primary function as a fuel reservoir, blubber has biomechanical, buoyancy, insulation, and thermoregulatory properties (Beck et al., 2000; Liwanag et al., 2012; Mellish et al., 1999). Blubber thickness varies depending on the species and life cycle, from 1 mm in new‐born pinnipeds to up to 50 cm in bowhead whales (*Balaena mysticetus*) (Iverson & Koopman, 2018). Blubber volume also varies dramatically with season (Beck et al., 2000; Fedak & Anderson, 1982; Sparling et al., 2006), but there is no evidence that such fluctuations have a negative impact in the animals’ health. Blubber appears to be vertically stratified or graded, such that the outer layer has a greater role in insulation, while the inner layer is more metabolically active, and the middle layer is more expandable as fat reserves change in size (Guerrero & Rogers, 2017; Robinson et al., 2018; Tverin et al., 2019). Moreover, there is a thermal gradient from ambient temperature to core temperature, where skin temperature is cooler than the muscle (Worthy, 1991). Marine mammals’ thick subcutaneous adipose tissue, rapid volume fluctuation, and differential vertical features may thus impact on tissue oxygen tension, particularly during its periodic rapid expansion, irrespective of the presence or absence of metabolic complications.

Grey seals (*Halichoerus grypus*) undergo periodic massive and rapid changes in subcutaneous adipose tissue mass during their life cycle: fatness ranges from 15 to 55% of body composition (Beck et al., 2000, 2003; Fedak & Anderson, 1982; Hall & McConnell, 2007; Sparling et al., 2006). During the molt in spring, food intake is reduced and body fat declines. After molting, adult female fat stores increase over summer and autumn to approximately ~34% of body mass (Beck et al., 2000). Their fat reserves decline again rapidly during the ~18 day suckling period, when the female simultaneously fasts onshore and produces high‐fat milk (Beck et al., 2003; Fedak & Anderson, 1982). Pups are born at about 15 kg with a thin layer of fat, but rapidly increase mass, mostly as blubber, at a typical rate of ~1.5–2.5 kg/day (Fedak & Anderson, 1982) and they wean at ~40% body fat (Beck et al., 2003; Hall & McConnell, 2007; Sparling et al., 2006). Lactation ends abruptly when the mother returns to sea. Pups then undertake a prolonged fast of ~10 days to 1 month before they go to sea for the first time. Fat percentage declines from ~40 to ~15% in the first year (Beck et al., 2003; Hall & McConnell, 2007; Sparling et al., 2006). After their first molt, juveniles build up their blubber fat once more. At some points in their annual cycle, seals’ adiposity thus reaches similar levels to those observed in obese humans (Wang et al., 1999). However, there are no studies on how seals cope with the challenges of rapid adipose tissue expansion in terms of oxygen availability.

Here, we measured blubber Po_2_ in juvenile grey seals. We tested the hypothesis that falls with increasing body fatness. We also investigated, where possible, whether Po_2_ drops with tissue expansion during weight gain. In addition, we hypothesized that Po_2_ would be lower in the outer blubber layers and at lower temperatures due to vasoconstriction. These findings will shed light on the potential constraints on maintaining adequate oxygenation throughout blubber in an animal with thick subcutaneous adipose tissue, and natural, rapid adipose tissue expansion.

## 2 METHODS

### 2.1 Ethical Approval

Capture and sampling of juvenile grey seals were conducted by licenced personnel under Home Office project licence 70/7806 in compliance with Animal (Scientific Procedures) Act (ASPA) 1986 and the EU directive on the protection of animals used for scientific purposes (2010/63/EU). This work was also approved by Abertay University Ethics panel (approval EMS825) and complies with the Physiological Reports animal ethics checklist.

### 2.2 Animal capture and husbandry

Twelve healthy, wild, juvenile grey seals were included in this study. They were captured from the Isle of May or Culbin Sands, Scotland. Body mass was under 60 kg for all animals, suggesting they were juveniles (Hall & McConnell, 2007). Seals were sedated with a mass apportioned intramuscular dose of 0.5–1.0 mg/kg midazolam (10 mg/2 ml, Hypnovel, Roche) and transported to the seal holding facility at the Sea Mammal Research Unit (SMRU). A maximum of nine seals at a time were kept in four inter‐connected seawater pools (Sparling et al., 2006) where they had free movement between pools and haul‐out areas. Monthly seawater quality assessments were carried out by a UKAS approved laboratory (Tayside Scientific Services, Dundee). Seals remained captive for a maximum of 7 months in total before being released into their natural habitat. Immediately prior to transport, animals were sedated with midazolam, transported, and released in their respective capture location.

Initial and daily health assessments of animals’ general condition ensured that only healthy individuals were included in the study. Animals were fed daily with 2.8 ± 1.2 kg of herring (occasionally sprat, hake, or sandeel) (Marine Nutrition, Grimsby, England) supplemented with vitamins (1 Aquavits and 300 mg ferrous gluconate per day, supplied by the International Zoo Veterinary Group).

### 2.3 Experimental design

Animals were sampled for this study after they had molted to ensure that blood flow changes during hair replacement would not affect comparisons of the same animal at different masses (Paterson et al., 2012). Animals were fasted overnight and isolated for sampling. Intramuscular midazolam and intravenous 1 mg/kg ketamine (Ketamidor, Chanelle, VET Ltd) or 0.1 mg/kg zoletil _100_ (50 mg/ml zolazepam and 50 mg/ml tiletamine, zoletil 100, Virbac) were used to sedate and anesthetize the animals, respectively. Dissolved oxygen levels (Po_2_) were measured in blubber tissue, at different blubber depths, on the dorsal flank in four female and eight male seals when they were in a mass gain trajectory. Four of them, one female and three males, were resampled after mass gain when blubber thickness had increased. Morphometric parameters and blubber depth were measured. Peripheral blood oxygen saturation (SpO_2_), heart rate (HR), respiration events, blubber hemoglobin volume, and relative hemoglobin oxygenation were measured during sampling to account for variation in oxygen supply and delivery.

### 2.4 Morphometrics and fatness estimation

Length was measured from nose to tail, and girth was taken around the maximum axillary region with a measuring tape (± 0.1 cm) (Hall & McConnell, 2007). Mass was measured using a floor scale to the nearest 0.2 kg (Avery Weigh ‐Tronix, Smethwick, UK). Once morphometrics were taken, animals were moved into an indoor area for instrumentation and measurements. Two measurements of blubber thickness (hereafter, total blubber depth) from the right flank were taken using a 5MHz probe of B‐Mode ultrasound (CS‐3000, Diagnostic Sonar, Livingstone, UK).

Fatness was estimated using a seal‐specific metric, termed *LMD* index, as shown in Equation (1) (Ryg et al., 1990):

$$LMD=4.44+5693\sqrt{L/M}\cdot d$$

where *LMD* is an index for blubber fat content, *L* was the length (m), *M* the mass (kg), and *d* the total blubber thickness (m).

Percentage blubber fat content was calculated as an estimation of fatness, assuming seals only store fat in blubber (Nordoy & Blix, 1985; Reilly & Fedak, 1990). We have not used BMI to avoid the health implication inferences of fat accumulation that this metric has for humans.

### 2.5 Arterial blood oxygen supply

Breathing rate, SpO_2_, and HR were monitored during sampling to maintain an appropriate plane of anesthesia. Spontaneous breath holding is common in sleeping and anesthetized grey seals and this may influence local tissue oxygen levels (Fedak et al., 1988; Mortola & Lanthier, 1989). Nostril and thoracic cage movement were filmed with a GoPro throughout the process allowing the time of each inhalation to be recorded from the videos and synchronized across instruments. Breath hold duration and time since last breath were calculated for each breath throughout the measurement period.

A pulse oximeter (VM‐2160, Viamed, West Yorkshire, UK) was placed in the rectum to record SpO_2_, as a surrogate of arterial blood oxygen saturation, and HR throughout sampling. SpO_2_ was used in statistical modeling to control for oxygen delivery to the blubber.

### 2.6 Local blubber blood supply

Relative changes in blood volume (total hemoglobin) and relative changes in the difference between oxyhemoglobin and deoxyhemoglobin (hereafter, hemoglobin oxygenation) were recorded using a custom‐made NIRS device (PortaSeal, Artinis Medical Systems, Einsteinweg, The Netherlands) (McKnight et al., 2019) to account for tissue‐specific blood oxygen changes in blubber throughout sampling as a result of potential vasoconstriction / dilation in local microvasculature. Light sources and detector were 28 mm (850 and 751 nm optode), 33 mm (852 and 751 nm optode), and 38 mm (851 and 752 nm optode apart), resulting in three different channel depth recordings. The NIRS device was placed on the shaved skin of the dorsal flank, near where the dissolved oxygen measurements were being recorded. The device was held in place by hand for at least 3 min, recording with a differential path length factor of 6 DPF at 50 Hz, with 1 s response time. Start and finish times for the recording were noted for data alignment with the other data sets. Each trace was visually inspected, and sudden large increments or decrements were removed from the analysis, as they were likely technical errors due to animal movement. Animals resampled after mass gain were not included in statistical analysis of the NIRS data because the recordings had light interference.

### 2.7 Blubber temperature and dissolved oxygen measurements

**FIGURE 1:**
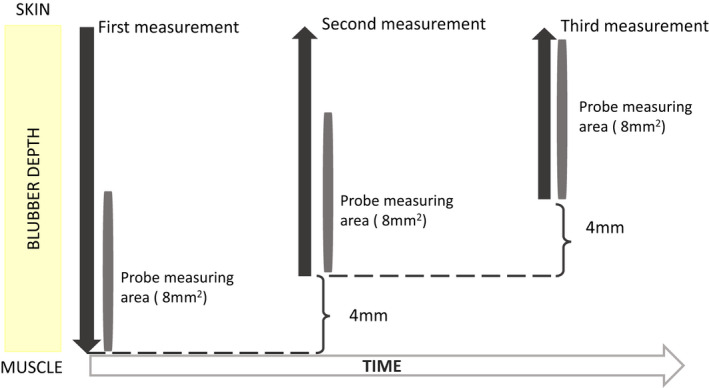
Diagram of how the Po_2_ measurements were taken from one animal at different depths. Dark grey arrows illustrate direction of probe insertion and retraction. Grey bars show the area over which the probe measured Po_2_ at each depth

Optical, temperature‐compensated oxygen probes (NX‐LAS‐8/OT/E, Oxford optronix, Abingdon, UK) were used to measure blubber Po_2_ and blubber temperature (T_b_). This pre‐calibrated probe measures Po_2_ using a ruthenium sensor that detects oxygen‐induced light quenching (Griffiths & Robinson, 1999), and averages over an area of 8 mm^2^. The accuracy in our measurement range (7–150 mmHg) was ±10%. The probe also records temperature (± 0.2°C), in addition to performing internal temperature compensation, over a 10–40°C range. Temperature and Po_2_ were recorded using LabChart Pro8 (AD Instruments, Sydney, Australia). Following the manufacturer’s advice, the calibration of the probe was confirmed by the OxyLite or OxyLite Pro device (Oxford optronix, Abingdon, UK) and an initial measurement of Po_2_ in air before each use. Measurements in air were always within the acceptable recommended range (145–170 mmHg). Based on the ultrasound measurements of total blubber depth and the length of the probe (8 mm), we calculated the depths at which the measurements would be taken (Figure 1). We maximized the number of measurements while avoiding overlap of more than 4 mm. The probe was then inserted through a 16G needle and placed to the deepest blubber depth, nearest the muscle. After 3–5 min of equilibration, the probe was kept in position for another 30 s to take the measurement. Stabilization was checked visually from the LabChart read out. Then, the probe was retracted by 4–6 mm to the next depth to take the next measurement. The process was repeated up to three more times depending on the total blubber depth of the animal. Start and finish times of the recordings and time of probe movement were noted to align the data with the other data sets. Depth of each measurement, taken as the middle point of the length of the probe (8 mm) was expressed as a percentage of total blubber depth (hereafter, depth percentage) to investigate if blubber depth affects Po_2_. A depth percentage of 100% would correspond to the blubber touching the muscle while 0% would be in contact with the skin.

### 2.8 Site of measurement

To investigate the effect of the position of the oxygen probe on the flank (inter‐site variability) and the depth of measurement (depth percentage), two probes were inserted simultaneously 33 ± 3 mm apart on the animals that were resampled after mass gain (*n*_animals_ = 4). After equilibration, 30 s of recording were averaged. Measurements were taken at different depths as described above (Figure 1).

### 2.9 Data processing

Data streams were synchronized to a 1 s resolution. SpO_2_ and HR data were recorded with a 4 s resolution. A linear extrapolation was applied to convert these data to 1 s resolution to align to the other data sets. Po_2_ and T_b_ measurements were acquired every second, with a 5 s rolling averaging. SpO_2_, HR, Po_2_, and T_b_ data were aligned by time with a 1 s resolution. NIRS data could not always be recorded simultaneously when an animal moved or while moving the oxygen probe. Therefore, it was processed separately, keeping the recorded frequency (50 Hz).

### 2.10 Statistics

Data analysis was performed in RStudio 4.0.0 (R Core Team (2018)). Data exploration was carried out as follows (Zuur et al., 2010). Briefly, outliers were identified by boxplots, and confirmed by Cleveland plots and Grubbs test. Outliers were only discarded when there was a strong rationale. For example, SpO_2_ values outside the 75–100% range were considered likely due to technical errors caused by animal movement displacing the probe. Po_2_ measurements <10 mmHg were not included, because visual observation of the probe time series showed a potential transient vasoconstriction reaction to the probe insertion. Distribution was explored visually using histograms and normal distribution was confirmed by Shapiro–Wilk tests. Data exploration was performed separately for each data set.

Linear mixed effect models (LME) from “*nlme”* package with fixed intercepts were used to investigate the associations between variables as described below. Animal ID was included as a random effect to account for repeated measurements from the same individual. Variable collinearity was assessed by variation inflation factor (VIF), and only variables with VIF <3 were included in each model. Models were individually validated visually by plotting the residuals versus fitted values and a qqplot. Model fit was assessed by R squared using “*piecewiseSEM”* package. For each variable included in the models selected by AIC, 85% confidence intervals were calculated to allow us to exclude biologically uninformative variables when 85% CI overlapped with zero (Arnold, 2010).

### 2.11 Arterial blood and local blubber oxygen supply

We first used all sampling occasions to investigate whether oxygen delivery varied during sampling. Stationarity of SpO_2_, local blubber blood volume, and hemoglobin oxygenation were tested with an augmented Dickey–Fuller test for each animal to identify if oxygen supply was stable during the measurement period. For blubber blood volume and hemoglobin oxygenation we only included the first time we sampled each animal and we excluded animal H due to lack of stability in Po_2_ (*n*_animals_ = 11).

### 2.12 Site of measurement

We then wanted to establish whether Po_2_ measurements are reproducible across different flank sites and depth (*n*_animals_ = 4). Site and depth percentage were included as explanatory variables for the Po_2_ measurements taken from two probes in tandem. Interaction between site and depth was included to represent the specific microenvironment at a given time.

### 2.13 Blubber temperature

To explore what changes occur in blubber temperature that may affect Po_2_, an LME including T_day_, depth percentage, LMD index, and the interaction between the three were used in the global model using “*dredge*,” a backward model selection tool. Goodness of fit was assessed by AICc. When weight of the best model was <0.9, models with delta AICc <2 were considered to have equal fit (Grubber et al., 2011). Animal E was not included in the analysis due to technical complications in the sampling. These reflected in lower temperature measurements of the probe that were detected as outliers and very influential in the model (blubber measurements <27°C and air measurements slightly lower compared to previous air reading on the same day). One additional outlier (T = 28.4°C) detected in the model residuals, was also removed, which improved the model fit.

### 2.14 Blubber Po_2_

To explore blubber Po_2_ variability, we used LME (*n*_animals_ = 10, *n*_observations_ = 31) with log transformation of Po_2_ before analysis. We then included our target variables, LMD index, and depth percentage as fixed effects in an LME. We also included SpO_2_ as a proxy of oxygen delivery, T_b_ which might affect hemoglobin download, and sex. A triple interaction between LMD index, depth percentage, and T_b_ was also included. Model exploration was carried out using “*dredge*” as described above. As outlined above, animal E was excluded due to technical complications. Additionally, animal H was discarded because Po_2_ measurements did not stabilize in 43 min.

To confirm if Po_2_ variability was related to tissue expansion rather than general fatness, we compared Po_2_ measurements before and after mass gain with a two‐sided paired *t*‐test in the animals for which we had a second measurement after mass gain (*n*_animals_ = 4, *n*_observations_ = 8).

## 3 RESULTS

### 3.1 Morphometrics and fat estimation

Characteristics of animals included in the final analyses are summarized in Table 1. Mass was 34.8–80 kg (49.9 ± 13.2 kg SD) and total blubber depth ranged from 10 to 27 mm (18 ± 6.3 mm SD). LMD, the fatness index used here, was 14.5–28.3% (20.4 ± 4.5%). Animals that were resampled increased mass at a rate of approximately 0.13 kg/day, with an absolute increase of 8.8–24.6 kg, ~35% increase in fatness, 7–15 mm increase of total blubber depth, and a maximum of 11 cm growth in body length over 4–6 months.

**TABLE 1:** Summary of animal characteristics (*n*_animals_ = 12). Animals were sampled once (*n*_animals_ = 7) or twice (*n*_animals_ = 4) after mass gain

Date	Animal	Sex	Weight (kg)	Length (cm)	Girth (cm)	Blubber depth (mm)	Blubber fat percentage (LMD index) (%)
07/05/2018	B	Female	38.6	116	88	19	23.2
07/05/2018	C	Female	34.8	119	80	11	16.0
07/05/2018	D	Male	47	127	88	15	18.5
07/05/2018	E	Male	40.6	119	83	12	16.1
07/05/2018	F	Male	42.2	115	89	16	19.5
18/07/2018	H	Male	64.4	132	106	26	25.6
18/07/2018	G	Male	47.6	123	95	20	22.7
06/12/2018	G	Male	60	134	103	28	28.3
18/07/2018	I	Female	37.8	112	88	12	16.2
22/08/2018	J	Male	59.4	128	102	20	21.2
06/12/2018	J	Male	68.2	132	106	27	25.8
18/07/2018	K	Male	39	123	89	10	14.6
19/07/2018	L	Female	36	113	85	12	16.9
06/12/2018	L	Female	60.6	122	102	25	24.6
21/06/2018	M	Male	48	126	94	11	14.6
06/12/2018	M	Male	80	131	113	26	23.4

### 3.2 Arterial blood oxygen supply

We expected local oxygen supply to be affected by breathing pattern, SpO_2_, and HR (Fedak et al., 1988). Therefore, we investigated to what extent these variables fluctuated within and between sampling. First sampling of animal G, for which we could not see nostril movements clearly from the footage, was excluded from this analysis.

**FIGURE 2:**
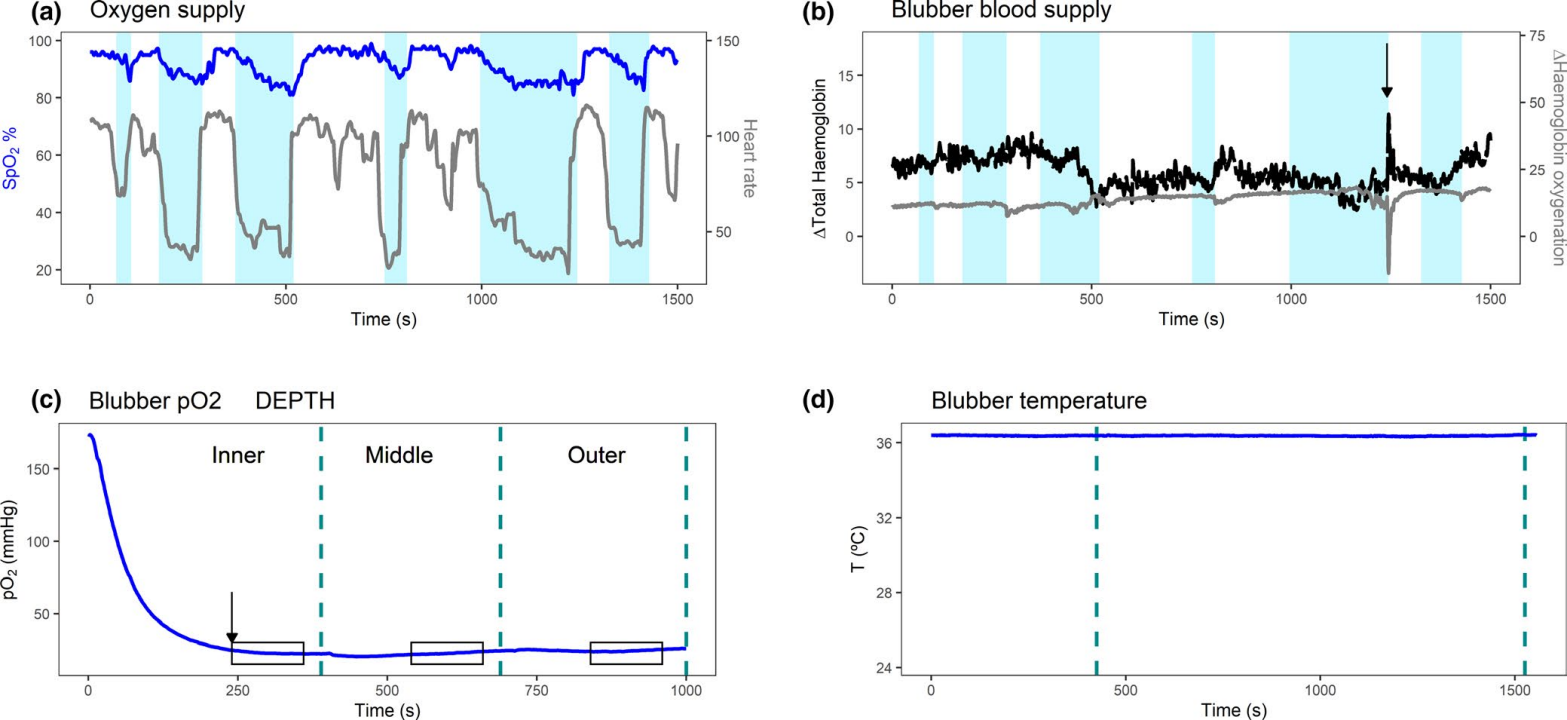
Example traces over time. (a) Oxygen supply: Breath holding events of over 30 s are shaded in light blue. SpO_2_ (left axis, top line in blue), heart rate (right axis, bottom line in grey). (b) Blubber blood supply: Breath holding events of over 30 s are shaded in light blue. Total blood volume is represented in black (left axis, top line) and hemoglobin oxygenation difference in grey (right axis, bottom line). Arrow indicates a sudden movement of the animal, probably due to a deep inhalation (c) Blubber Po_2_: Arrow indicates the end of the stabilization period. Dashed lines represent the movement of the probe to the next depth and boxes the duration over which the data were averaged for analysis. (d) Blubber temperature (left axis, top line in blue). Dashed lines represent the movement of the probe to the next depth

For breathing pattern, we analyzed a total of 2219 s from 11 animals, 3 of them repeated after mass gain. Animals showed a regular breathing pattern throughout sampling (Breath hold length median = 5 s), except for 127 breath holds of between 50–130 s and 5 breath hold events over 130 s (max = 309 s). Given long breath holds were eventual, the potential impact in Po_2_ was discarded and breath hold duration was not included when exploring the drivers for Po_2_. HR was analyzed from a total of 2219 s recording, and was very variable (74.3 ± 22.3 SD bpm, *n*_animals_ = 12, *n*_observations_ = 16). Visual observation suggested that long breath holds were associated with reduced HR (Figure 2a). However, there was no collinearity between breath hold duration and HR (VIF <3), likely because long breath holds were rare.

SpO_2_ was consistent between animals and throughout the sampling of each animal (90.2 ± 5.9 SD %, *n*_animals_ = 12, *n*_observations_ = 16), except for the first sampling of animals E, I, K, and L (Table 2). An example trace is shown in Figure 2a. Given that SpO_2_ was not stationary in some animals it was included in further analysis as an explanatory variable in blubber Po_2_ measurements.

**TABLE 2:** Output of augmented Dickey–Fuller test for systemic blood saturation (*n*_animals_ = 12), total hemoglobin (*n*_animals_ = 11), and hemoglobin oxygenation (*n*_animals_ = 11). Animal H and animals resampled after mass gain were not included in blubber hemoglobin analysis (NA)

		SpO_2_		Total hemoglobin		Hemoglobin oxygenation	
Date	Animal	Dickey–Fuller test	*p*‐value	Dickey–Fuller test	*p*‐value	Dickey–Fuller test	*p*‐value
07/05/2018	B	−6.7985	≤0.01	−5.5700	≤0.01	−5.4380	≤0.01
07/05/2018	C	−3.4256	0.05	−10.092	≤0.01	−6.1414	≤0.01
07/05/2018	D	−4.5414	0.01	−17.107	≤0.01	−8.1425	≤0.01
07/05/2018	E	−2.2864	0.46	−7.6322	≤0.01	−10.1500	0.01
07/05/2018	F	−4.0999	≤0.01	−3.3455	0.0627	−7.3087	≤0.01
18/07/2018	G	−4.3708	≤*0*.*01*	−4.6603	≤0.01	−2.9951	0.1544
06/12/2018	G	−6.5936	≤*0*.*01*	NA	NA	NA	NA
18/07/2018	I	−2.9499	*0.18*	−3.3604	0.0600	−3.5650	0.0358
18/07/2018	H	−6.0899	≤0.01	NA	NA	NA	NA
22/08/2018	J	−3.4324	*0.05*	−11.5860	≤0.01	−2.9182	0.1863
06/12/2018	J	−4.9117	≤*0*.*01*	NA	NA	NA	NA
18/07/2018	K	−2.1866	*0.50*	−4.9860	≤0.01	−5.3221	≤0.01
19/07/2018	L	−2.4067	*0.41*	−2.5541	0.3434	−5.4451	≤0.01
06/12/2018	L	−9.2874	≤0.01	NA	NA	NA	NA
21/06/2018	M	−9.1633	≤0.01	−4.2387	≤0.01	−5.9610	≤0.01
06/12/2018	M	−11.758	≤0.01	NA	NA	NA	NA

### 3.3 Local blubber blood supply

In order to assess oxygen delivery to the blubber, relative total hemoglobin and hemoglobin oxygenation difference changes over time were investigated in the first sampling (*n*_animals_ = 11) (McKnight et al., 2019). A total of 12,201 s of NIRS data were examined. An example trace is shown in Figure 2b. Relative blood volume maintained stationarity over the sampling period, except for animals F, I, and L (Table 2). Therefore, blubber blood supply was stable in most animals throughout sampling. For hemoglobin oxygenation, stationarity was confirmed by augmented Dickey–Fuller test, except for animals G and J (Table 2). Although relative total hemoglobin and relative hemoglobin oxygenation changes were not stable in all samplings, we did not have sufficient simultaneous NIRS measurements to include them as explanatory variables in the model for Po_2_.

### 3.4 Blubber Po_2_ measurements

Each animal was sampled at 1–4 depths, depending on their total blubber depth. A representative trace of blubber Po_2_ is shown in Figure 2a. Blubber Po_2_ ranged from 14.5 to 71.4 mm Hg (39.2 ± 14.1 SD mm Hg), with large inter‐animal variability (Table 3).

**TABLE 3:** Oxygen probe measurements of each animal at each depth percentage. Averages of Po_2_ and temperature (± SD) are shown in the final two columns

Date	Animal	Sex	Depth percentage	Po_2_ (mmHg)	Tb (ºC)
7/5/2018	B	F	37	57.3 ± 0.4	32.7 ± 0.2
7/5/2018	B	F	74	49.6 ± 0.9	34.8 ± 0.1
7/5/2018	C	F	45	50.2 ± 0.5	32.0 ± 0.6
7/5/2018	C	F	64	48.4 ± 0.5	32.8 ± 0.6
7/5/2018	D	M	47	37.2 ± 0.1	33.8 ± 0.1
7/5/2018	D	M	73	59.1 ± 0.4	33.8 ± 0.1
7/5/2018	E	M	33	20.8 ± 0.1	26.5 ± 0.1
7/5/2018	E	M	67	17.7 ± 0.9	26.0 ±0.3
7/5/2018	F	M	44	38.2 ± 0.6	32.7 ± 0.2
7/5/2018	F	M	75	36.8 ± 2.4	35.6 ± 0.1
18/7/2018	G	M	35	34.4 ± 0.1	34.6 ± 0.3
18/7/2018	G	M	55	40.5 ± 0.2	33.6 ±0.6
18/7/2018	G	M	80	38.9 ± 0.3	35.5 ± 0.0
6/12/2018	G	M	21	22.9 ± 0.1	30.0 ± 0.2
6/12/2018	G	M	43	14.5 ± 1.7	35.4 ± 0.0
18/7/2018	I	F	33	71.4 ± 0.6	34.5 ± 0.2
18/7/2018	I	F	67	69.3 ± 1.2	34.9 ± 0.1
22/8/2018	J	M	35	51.2 ± 0.1	31.2 ± 1.9
22/8/2018	J	M	55	42.2 ± 1.0	35.3 ± 0.1
22/8/2018	J	M	75	41.0 ± 0.8	35.2 ± 0.5
6/12/2018	J	M	26	23.2 ± 1.0	32.7 ± 0.6
6/12/2018	J	M	56	19.3 ± 3.1	32.7 ± 0.7
6/12/2018	J	M	85	23.0 ± 0.6	28.7 ± 0.3
18/7/2018	K	M	60	53.0 ± 0.3	34.3 ± 2.0
19/7/2018	L	F	33	31.9 ± 0.2	33.9 ± 1.0
19/7/2018	L	F	67	28.6 ± 0.2	34.8 ± 0.7
6/12/2018	L	F	36	31.4 ± 0.4	33.7 ± 1.2
6/12/2018	L	F	60	27.7 ± 6.8	35.2 ± 0.4
6/12/2018	L	F	84	27.7 ± 0.8	35.4 ± 0.2
21/6/2018	M	M	64	49.6 ± 0.5	35.4 ± 0.1
6/12/2018	M	M	38	36.8 ± 2.6	35.1 ± 0.1
6/12/2018	M	M	62	27.8 ± 5.8	35.7 ± 0.1
6/12/2018	M	M	85	32.1 ± 3.7	35.5 ± 0.1

### 3.5 Site of measurement

In the four animals for which we tested the effect of site of the measurement (LME: AIC = 147.5, *R* ^2^_marginal_ = 0.08, *R* ^2^_conditional_ = 0.7, *n*_animals_  = 4, *n*_observations_  = 22), neither site (estimates = −8.57, df = 15, *t*‐value = −1.53, *p* = 0.1466, 85% CI [−16.3, −0.89]), nor the depth percentage (estimates = −0.12, df = 15, *t*‐value = −1.77, *p* = 0.0964, 85% CI [−0.21, −0.03]) significantly affected Po_2_. More importantly, the interaction between site and depth percentage was not significant (estimates = −0.14, df = 15, *t*‐value = 1.44, *p* = 0.1693). We were therefore able to eliminate sampling site as a major source of variability in Po_2_ measurements in this study.

### 3.6 Blubber temperature measurements

T_b_ was similar between samplings (T_b_ = 34.3 ± 1.9°C SD), despite a 15–22°C range in ambient temperature (T_day_). T_b_ had a normal distribution (Shapiro test: W = 0.89 *p* = 0.061) and model residuals complied with normality and heterogeneity. The models to explain T_b_ selected by dredge and based on AICc are shown in Table 4. The best model did not include any of the parameters, but the next best model was almost identical and included LMD (LME: estimates = −2.39, df = 17, *t*‐value = −2.49, 85% CI [−3.74, −1.05]), T_day_ (LME: estimates = −2.70, df = 17, *t*‐value = −2.37, 85% CI [−4.31, −1.10]), and the interaction among both (LME: estimates =0.12, df = 17, *t*‐value =2.55). The following selected models had a very weak fit. However, the best model only explained 26% of the variability in T_b_. Animal identity did not explain any of the variability in T_b_.

**TABLE 4:** LME models for drivers of blubber temperature (*n*_animals_ = 10, *n*_observations_ = 32)

Model	df	AICc	Delta	Weight	*p*‐value variables model				*R* ^2^_marginal_	*R* ^2^_conditional_
					LMD	Depth percentage	T_day_	LMD^*^ T_day_		
1	3	129.1	0.00	0.283	–	–	–	–	–	–
2	6	129.2	0.03	0.278	0.0233	–	0.0301	0.0206	0.26	0.26
3	4	129.9	0.76	0.193	–	–	0.1902	–	0.06	0.06
4	4	130.6	1.45	0.137	–	0.2935	–	–	0.04	0.04
5	4	131.0	1.89	0.110	0.4003	–	–	–	0.03	0.03

### 3.7 Drivers of blubber Po_2_

**FIGURE 3:**
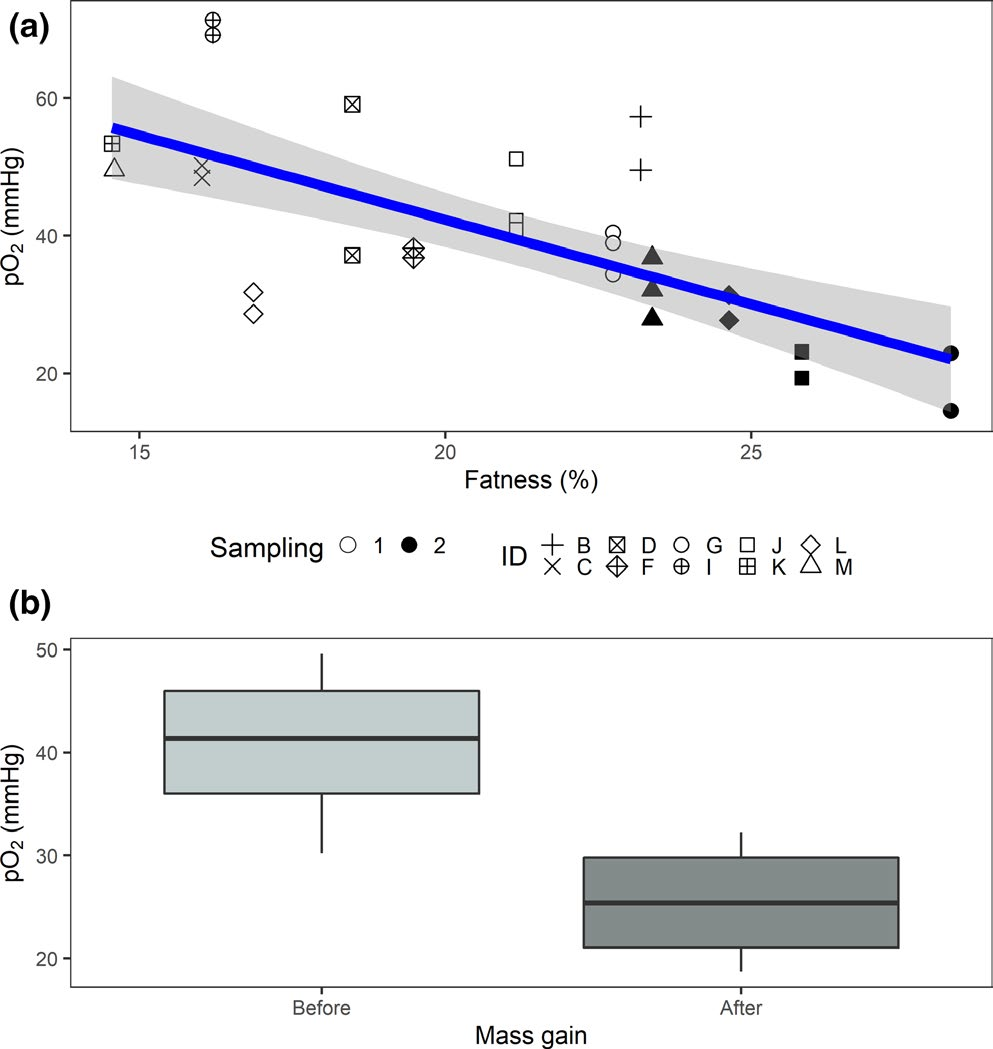
Relationship between absolute Po_2_ values and (a) fatness (LMD), where repeated measurements correspond to different depths for each individual. Smooth line (blue) from best model output for fatness with standard error (grey shadow). Resampling measurements after mass gain are represented with solid symbols; and (b) Comparasion between Po_2_ values before and after mass gain for those animals that were resampled after blubber tissue expansion (*n*_animals_ = 4)

For all animals, blubber Po_2_ had a normal distribution (Shapiro test: W = 0.97, *p* = 0.4718). An LME including T_b_, depth percentage, sex, LMD index, and SpO_2_ and a triple interaction between LMD, T_b_, and depth percentage were used as the global model in dredge. The models to explain Po_2_ based on ΔAICc <2 are shown in Table 5. All models retained the LMD index. Sex and T_b_ were retained only in one model each, but were not a significant explanatory variable in each case. Neither depth percentage nor any interactions was retained. The best model (model 1, Figure 3a) had almost double the weight of the next best model and was also the most parsimonious because it only retained fatness as the explanatory variable. Fatness was strongly and negatively associated with Po_2_ (estimate = −0.06, df = 20, *p* < 0.0001, 85% CI [−0.08, −0.05]).

**TABLE 5:** LME models for drivers of blubber Po_2_ (*n*_animals_ = 10, *n*_observations_ = 31)

Model	df	AICc	Delta	Weight	*p*‐value variables model					*R* ^2^_marginal_	*R* ^2^_conditional_
					LMD	Depth percentage	Sex	SpO_2_	T_b_		
1	4	11.4	0.00	0.515	<0.0001	–	–	–	‐	0.51	0.63
2	5	12.6	1.23	0.278	0.0001	–	–	–	0.2117	0.51	0.67
3	5	13.2	1.82	0.207	<0.0001	–	0.3852	–	–	0.55	0.74

The subset of animals that was sampled before and after mass gain followed a normal distribution (Shapiro test: W = 0.96, *p* = 0.8482). The initial percent blubber content (14.6–22.7%) was lower than in animals after mass gain (23.4–28.3%) in all cases (Table 3). One animal’s LMD index estimate (G: 22.7%) in the first sampling was similar to the LMD index from M in the second sampling (23.4%). Three of the four animals showed a decline in Po_2_ measurements after mass gain, while L remained similar (Table 3). In this very small subset of animals resampled after mass gain, Po_2_ after blubber tissue expansion was not significantly lower than before, suggesting low power to detect a difference in Po_2_ of ~37% in such a small sample size (paired *t*‐student: *t* = 3.17811, df = 3, *p*‐value = 0.0502, 95% CI [−0.02, 30.4]) (Figure 3b).

## 4 DISCUSSION

To the best of our knowledge, this is the first study to measure Po_2_ in blubber of a marine mammal. Our Po_2_ measurements in juvenile grey seals (15.4–71.4 mmHg) are within the same range of those reported in adipose tissue from rodents and humans (Cifarelli et al., 2020; Goossens et al., 2011; Pasarica et al., 2009; Rausch et al., 2008; Vink et al., 2017; Ye et al., 2007). Although we did not measure metabolic health here, because there are not well‐defined metrics to do so in seals, there is no evidence that seals suffer any metabolic complications from large fat depots (Houser et al., 2013; Khudyakov et al., 2019), yet our data show they have similar oxygen availability in subcutaneous adipose tissue to other species that do develop complications from fat accumulation, which are often attributed to low oxygen levels (Hosogai et al., 2007; Ye et al., 2007). We did not record very low levels of Po_2_, except in one instance, which we attributed to transient vasoconstriction in response to the introduction of the probe where Po_2_ transiently reached 1.5 mmHg. Our measurements were taken in tidally breathing seals in air under anesthesia and therefore do not reflect what may happen to blubber Po_2_ during routine dives. Po_2_ measurements in arterial blood in diving elephant seals showed that seals experience repeated and extended low arterial blood Po_2_ (Meir et al., 2009). Additionally, NIRS measurements on blubber of voluntarily diving harbor seals indicate vasoconstriction in blubber that may result in low Po_2_ (McKnight et al., 2019). These results taken together, suggest that seals might face much lower tissue Po_2_ levels than recorded here.

Hypoxia is often defined as oxygen tension lower than 10 mmHg (Trayhurn, 2013), which comes from cancer studies, in which tumor oxygen levels are charcterized in comparison to “healthy” tissue. “Normal” oxygen levels will differ depending on tissue requirements. It may not be appropriate to generalize this 10 mmHg threshold, especially for tissues with low metabolic requirements, such as adipose, which may still have adequate oxygen levels for their demands even when levels drop this low. Moreover, alterations in the molecular pathways are unlikely to be triggered by a strict cutoff value (Wood et al., 2011) and will depend on the length of exposure to reduced oxygen levels. In this study, we can not infer whether the observed Po_2_ levels induce hypoxia responsive pathways at a molecular level. However, downstream responses to hypoxia, such as an increase of HIF 1α have been reported at similar Po_2_ levels to our measurements (Lu et al., 2016; Rausch et al., 2008).

Consistent with rodent studies (Rausch et al., 2008; Ye et al., 2007) and some human research (Kabon et al., 2004; Pasarica et al., 2009) we found a strong significant negative relationship between Po_2_ and fatness (Figure 3a). In addition, we found a ~37% drop in Po_2_ before and after fattening in animals that we resampled after mass gain (Figure 3c). The comparison was only in four animals, likely lacking the power to detect any statistically significant differences. Studies with a larger sample size and bigger changes in fatness are needed. The measurements here reported will help in determining an statistically powered sample size. Nevertheless, our results support the theory that adipose tissue expansion lowers oxygen levels, and extend this to include grey seals, which naturally undergo large and rapid natural fat deposition. Grey seals may thus face oxygen restriction in blubber, not only during diving (McKnight et al., 2019; Meir et al., 2009), but also during fattening.

Although the animals here were in a mass gain trajectory, we were unable to explore the full range of fatness that wild seals experience during their life cycle. For example, the maximum blubber thickness of our study was 28 mm (Table 1), but weaned pups can reach up to 40 mm blubber thickness (Hall & McConnell, 2007; Hall et al., 2001). Juvenile seals are at their leanest point of life, other than the immediate neonatal period, with on average only ~12% body fat (Hall & McConnell, 2007). Moreover, the rate of mass gain in the animals resampled here (0.13 kg/day) was lower than the ~1.5–2.5 kg/day that grey seal pups experience during suckling (Fedak & Anderson, 1982; Mellish et al., 1999). We speculate that Po_2_ could become very low during extreme tissue expansion, and seals’ blubber could experience oxygen restriction during key life stages, even when resting with access to air.

Interestingly, low Po_2_ is not only a result of increased fat deposition, but can also drive the process. Indeed, a mice model of pseudohypoxia shows greater mass gain rate than the wild type (Michailidou et al., 2015). Consistently, multiple transient hypoxic events during adipocyte differentiation enhances triglyceride accumulation, insulin sensitivity, and antioxidant gene expression in mature adipocytes (Lu et al., 2016). If similar downstream molecular responses to low Po_2_ occur in seals, they may experience positive feedback during fat deposition phases of their life cycle as Po_2_ in blubber decreases, which could be augmented further if blubber becomes hypoxic during episodic breathing and apnea while resting on land (Mortola & Lanthier, 1989).

It is important to be cautious in inferring implications of Po_2_ measurements from studies on different species because circumstances leading to tissue expansion, their molecular pathways and health implications differ (Lempesis et al., 2019). Therefore, further studies specifically investigating blubber oxygen demands in relation to its metabolic requirements, and investigation of hypoxia molecular markers, such as HIF 1α, are needed to establish whether the lower Po_2_ measurements in this study are reflective of an hypoxic environment and have metabolic consequences in seals.

Despite a decrease in Po_2_ with overall fatness, blubber Po_2_ remained similar across blubber depth in any given animal. The consistency in Po_2_ across depth can be explained if the balance of oxygen consumption to delivery is maintained throughout blubber depth, at least in leaner individuals and when animals are breathing tidally and in their thermoneutral zone. There are currently no studies on seal blubber vascularity to suggest if blood supply varies with depth or tissue expansion. Biopsy samples of inner blubber appear to contain more blood than outer blubber (visual personal observation), but this may be a result of perfusion rather than vascularity *per se*. Vascularity in bottlenose dolphins (*Tursiops truncatus*) shows clear stratification, where superficial blubber is significantly less vascularized than deep blubber (McClelland et al., 2012). We speculate that inner blubber in grey seals is also more vascularized, but this increase in delivery capacity is counterbalanced by increased oxygen consumption. However, we cannot rule out that Po_2_ differences may develop across blubber depth in animals with larger fat depots. We also cannot rule out that depth differences may occur under more challenging conditions that result in localized vasoconstriction, such as colder ambient temperatures and when animals are active, diving, or undergoing episodic apnea on land. Depth of measurement therefore needs to continue to be considered in future studies.

Similar to studies in human adipose tissue (Kabon et al., 2004; Pasarica et al., 2009), and consistent with previous grey seal studies (Worthy, 1991), blubber temperature measurements ranged from 30.7 to 36.6°C and were constant throughout sampling (Figure 3b). These T_b_ measurements were below generally accepted normothermia (~37°C). This finding might be important for in vitro studies in which tissue is typically cultured at 37°C (Bennett et al., 2017; Robinson et al., 2018).

Surprisingly, we found no significant thermal gradient across blubber depth. This contrasts with Worthy (1991) study, where they found higher temperatures near the muscle compared to near the skin. Moreover, the thermal gradient continued through the muscle, regardless of blubber depth. Consistent with previous studies, in which ambient temperature‐related thermal gradients were reported, we found a weak association with ambient temperature, LMD index, and their interaction (Table 4) (Liwanag et al., 2012; Paterson et al., 2012). The significant association with LMD and the interaction between the explanatory variables suggest that temperature gradient might be more evident in animals with thicker blubber. Thermal gradients across blubber depth may thus be present in fatter animals. Moreover, our sampling was carried out in controlled, indoor facilities, minimizing the range of temperatures that animals were exposed to. In fact, the ambient temperature was well within seals’ thermo‐neutral zone (−7 to 23°C) (Hansen & Lavigne, 1997). Moreover, seals’ surface temperature changes with ambient temperature during life events such as molting and pregnancy, depending on body site and as a result of peripheral vasoconstriction (Chaise et al., 2019; Liwanag et al., 2012; Paterson et al., 2012). Therefore, blubber temperature may be less stable than seen here at different life stages and under different environmental conditions.

Interestingly, our results suggest that temperature does not influence Po_2_ when blubber is between 30.7 and 36.6°C (Table 5). Future studies in extreme temperature conditions and wider range of blubber thickness would shed light on the impact of temperature in blubber oxygenation.

Grey seals regularly hold their breath for 2–3 min while resting or sleeping (Knopper & Boily, 2000). During anesthesia, spontaneous breath holds are more common (Mortola & Lanthier, 1989). As expected, occasional longer breath holds were accompanied by a decrease in heart rate and systemic blood saturation here (Figure 2a). However, it was not the aim of our study to analyze the potential effect of breath holding on blubber oxygenation and we do not have enough data to perform a robust analysis from the few apnea events observed. During our study, the median duration of the breath holds was 5 s and therefore we assumed that the breathing pattern would not affect our blubber Po_2_ measurements. Moreover, since large breath holding events are reflected in SpO_2_, it is this variable rather than breathing frequency that we included in the main model. However, arterial blood oxygen saturation did not explain any of the variability in Po_2_ (Table 5) and remained relatively constant during our sampling (Table 2). The values were consistent with other species breathing in air and reflect that the animals were tidally breathing (Meir et al., 2009). Marine mammals routinely face peripheral vasoconstriction during diving, which includes reduction of blood volume and oxygenation in blubber throughout each dive (McKnight et al., 2019; Meir et al., 2009). As our sampling was performed when animals were breathing air, we cannot extrapolate the effects of SpO_2_ on blubber Po_2_ during diving or other large changes in SpO_2_.

To account for local blubber perfusion variations that might occur during sampling, we simultaneously sampled blubber blood volume changes and hemoglobin oxygenation differences with NIRS. This technology relies on the absorption of light by hemoglobin. It requires a constant pressure applied to the skin to hold the device in place to avoid interference from environmental light and maintain the consistent measurement depth. Unfortunately, movement of the animal during the recordings introduced artifacts in this dataset (Figure 2b) and there were not enough simultaneous NIRS data with the oxygen probe to be included in the main Po_2_ analysis. Visual inspections of the data set showed little variation in blood volume. Occasional small decreases most likely correspond to transient vasoconstriction events. Augmented Dicker–Fuller test confirmed that oxygen supply (i.e., SpO_2_ and NIRS oxygenation data) was constant over our sampling period (Table 2) and the site of measurement test showed that Po_2_ did not differ between site of sampling in this study. Therefore, we eliminated oxygen supply and site of sampling as sources of variation in blubber Po_2_ measurements in our data. We cannot rule out that other sites or other measurement conditions may show variation in oxygen supply. Of note is that fluctuations in SpO_2_ were not concordant with those in NIRS traces in most cases (Figure 2a,b), suggesting localized tissue O_2_ delivery and consumption are decoupled from systemic supply.

Anesthesia alters hemodynamic parameters (Afshar et al., 2005). Although we tried to standardize doses and timing across sampling, animals respond differently to the anesthesia and that could have affected our results. Moreover, we observed a 2–3 min acute decline in Po_2_ after the initial insertion of the probe. Although retraction of the probe to the next blubber depth point of measurement was not associated with any obvious changes, movement of the probe could be a potential cause for vasoconstriction in our sampling. Simultaneous measurements of perfusion would be useful in this type of study to account for technical and natural oxygen supply fluctuations that might affect Po_2_, but are difficult to achieve in practice since the NIRS device cannot remain in place over the probe site. Development of implantable or noninvasive Po_2_ probes would help to overcome this issue.

## 5 CONCLUSIONS

We found that dissolved oxygen levels in blubber of healthy juvenile seals at rest are similar to those in other mammals, despite their large fat depots. Blubber Po_2_ is strongly negatively associated with fatness in these animals. Despite oxygen depletion, the balance of oxygen consumption to delivery appears to be mantained across blubber depth. This paper contributes to the understanding of oxygen management in adipose tissue expansion in mammals and expands these observations to a species that undergoes natural, rapid fat deposition during key life history stages. However, the metabolic implications of oxygen restriction need further investigation to determine if blubber experiences hypoxia during extreme fattening or diving. To enhance our understanding of the implications of low blubber oxygenation in fatter seals, studies on adipose tissue vascularity, cell metabolism, and function during tissue expansion are important next steps.

## CONFLICT OF INTERESTS

The authors declare they have no competing interest to disclose.

## AUTHOR CONTRIBUTIONS

Conception or design of the work: LO, KAB, JCM, SEM, RM, AH, and JR. Data acquisition: LO, JCM, SEM, and RM. Data analysis or interpretation: LO, KAB, JCM, AH, and JR. Drafting the work or revising it critically for important intellectual content: LO, KAB, JCM, SEM, RM, AH, and JR.

## Data Availability

Data are available via FigShare https://doi.org/10.6084/m9.figshare.14743128.
